# Efficacy of ‘Tailored Physical Activity’ or ‘Chronic Pain Self-Management Program’ on return to work for sick-listed citizens: design of a randomised controlled trial

**DOI:** 10.1186/1471-2458-13-66

**Published:** 2013-01-23

**Authors:** Lotte Nygaard Andersen, Birgit Juul-Kristensen, Kirsten Kaya Roessler, Lene Gram Herborg, Thomas Lund Sørensen, Karen Søgaard

**Affiliations:** 1 Institute of Sports Science and Clinical Biomechanics, Faculty of Health Sciences, University of Southern Denmark, Odense, Denmark; 2 Department of Psychology, Faculty of Health Sciences, University of Southern Denmark, Odense, Denmark; 3 Senior Citizen and Health Department, Social and Health Affairs, Municipality of Sonderborg, Sonderborg, Denmark; 4 Regional Hospital, Avannaa, Ilulissat, Greenland, Denmark

**Keywords:** ‘Tailored Physical Activity’, ‘Chronic Pain Self-Management Program’, ‘Return to work’, ‘Musculoskeletal disorders’, Pain

## Abstract

**Background:**

Pain affects quality of life and can result in absence from work. Treatment and/or prevention strategies for musculoskeletal pain-related long-term sick leave are currently undertaken in several health sectors. Moreover, there are few evidence-based guidelines for such treatment and prevention. The aim of this study is to evaluate the efficacy of ‘Tailored Physical Activity’ or ‘Chronic Pain Self-Management Program’ for sick-listed citizens with pain in the back and/or the upper body.

**Methods:**

This protocol describes the design of a parallel randomised controlled trial on the efficacy of ‘Tailored Physical Activity’ or a ‘Chronic Pain Self-management Program’ versus a reference group for sick-listed citizens with complaints of pain in the back or upper body. Participants will have been absent from work due to sick-listing for 3 to 9 weeks at the time of recruitment. All interventions will be performed at the ‘Health Care Center’ in the Sonderborg Municipality, and a minimum of 138 participants will be randomised into one of the three groups.

All participants will receive ‘Health Guidance’, a (1.5-hour) individualised dialogue focusing on improving ways of living, based on assessments of risk behavior, motivation for change, level of self-care and personal resources. In addition, the experimental groups will receive either ‘Tailored Physical Activity’ (three 50-minute sessions/week over 10 weeks) or ‘Chronic Pain Self-Management Program’ (2.5-hours per week over 6 weeks). The reference group will receive only ‘Health Guidance’.

The primary outcome is the participants’ sick-listed status at 3 and 12 months after baseline. The co-primary outcome is the time it takes to return to work. In addition, secondary outcomes include anthropometric measurements, functional capacity and self-reported number of sick days, musculoskeletal symptoms, general health, work ability, physical capacity, kinesiophobia, physical functional status, interpersonal problems and mental disorders.

**Discussion:**

There are few evidence-based interventions for rehabilitation programmes assisting people with musculoskeletal pain-related work absence. This study will compare outcomes of interventions on return to work in order to increase the knowledge of evidence-based rehabilitation of sick-listed citizens to prevent long-term sick-leave and facilitate return to work.

**Trial registration:**

The trial is registered in the ClinicalTrials.gov, number NCT01356784.

## Background

Musculoskeletal pain causing sick-leave is multifactorial and may or may not have a negative effect on work participation. Sick leave has serious consequences for the individuals who take sick leave due to their pain, and it is fundamental for them to find ways to prevent or reduce their pain or, at least learn to cope with it [1].

Patient-oriented rehabilitation among individuals with work disability is a major challenge for municipalities in Denmark as they have principal responsibility for this function. In 2008 and 2009 respectively there were 687 and 757 new applications for sickness benefits due to disorders in the back and upper body in the Sonderborg Municipality. These disorders often include complex and diffuse symptoms that, over time, can result in chronicity and loss of function. Pain-related sick leave, particularly amongst younger workers, is often based on symptoms that are rarely consistent with a specific disease [2].

A study has shown that the interaction between the characteristics of the sick-listed, the company they are employed by and the municipality has important influence on return to work. In addition, economic conditions and sickness absence policies in society influence the number and length of absences, as well as the risk of being dismissed [3].

Individual factors, such as age, obesity, smoking, manual materials handling, lack of job control, lung restriction and reduced work ability have all been shown to be risk factors for sick leave [4]. Poorly perceived health status is associated with an extended duration of sick leave, and self-perceived health status can also predict return to work for persons with musculoskeletal complaints [5,6].

A systematic review of interventions for work-related complaints in the arm, neck and shoulder has shown limited evidence for the efficacy of physical exercises in patients with nonspecific work-related upper extremity musculoskeletal disorders compared with no treatment or as add-on treatment. Currently, there is no scientifically reported difference in the effects of various types of physical exercises on pain and pain coping [7]. Nor have solution-focused interventions delivered by psychologists been more effective than ‘treatment as usual’ for return to work or improvement in perceived health [8].

Treatment and prevention schemes for back and the upper body pain are common components of health care systems, but both lack evidence-based guidelines. The frequently incorporated elements include physical activity, diverse bio-psychosocial treatments, such as: manual therapy, electro-therapy, acupuncture, ergonomic guidance, cognitive behavioral therapy and/or patient education.

In this study tailored physical activity is the primary strategy for increasing the proportion of people on sick leave who return to work. Physical activity interventions involving strengthening exercises have been tested among various occupational groups to enhance their physical capacity and have proven to be effective in reducing pain and improving muscular strength [9,10]. Moreover, greater functional capacity as measured by cardiorespiratory fitness is related to increased quantity of work performed, and a higher level of cardiorespiratory fitness is related to a less effort required to perform the same work [11].

A systematic review of the effectiveness of community-based and workplace-based interventions to reduce musculoskeletal-related sickness absence and job loss concluded that no single intervention is more effective than another whether it be exercises or behavioral change. In addition, there was evidence that effort-intensive interventions were no more effective than simple interventions [12].

In this study the active comparator arm is a patient education programme called the “Chronic Pain Self-Management Program” (CPSMP). It was designed to enhance a person’s ability to cope with pain and it was developed through collaboration between Stanford University in California, USA and McGill University in Montreal, Canada. Use of this standardised programme requires education and certification from The Danish Committee for Health Education, which is the authority in Denmark with a licensed agreement with Stanford University. Previous studies of the effect of CPSMP have shown that participants experience, amongst other effects, less pain, improved mental health, increased level of daily activity, enhanced quality of life, greater confidence in their abilities and increased power to act compared with waiting list patients [13]. In 2010, implementation of the CPSMP commenced in Denmark and currently, 71 municipalities are licensed by The Danish Committee for Health Education to run this and related patient education programmes for people who have chronic diseases [14].

In spite of the large scale of the programme, it was stated in a report on patient education by the Danish Centre for Health Technology Assessment, that there is a lack of interdisciplinary research providing knowledge about the effects of patient education on sick leave. It is unknown whether further implementation of standardised programmes is warranted, given the level of scientific knowledge currently available [15].

Using a randomised controlled trial design, the aim of this study is to evaluate the efficacy of ‘Tailored Physical Activity’ (TPA) or ‘Chronic Pain Self-Management Program’ (CPSMP) versus a reference group (REF) on return to work. The intervention in each study arm is carefully chosen on the basis of former evidence-based studies that have shown effectiveness [9,10,16,17].

The current study is based on the hypothesis that TPA in the short term is superior in effect, while the outcome of CPSMP depends on behavioral changes and therefore may have a long-term effect. Therefore, outcome evaluations will be performed immediately after the end of the TPA intervention, 3 months (short-term) after baseline, and 12 months (long-term) after baseline.

## Methods

### Study design

**Figure 1:**
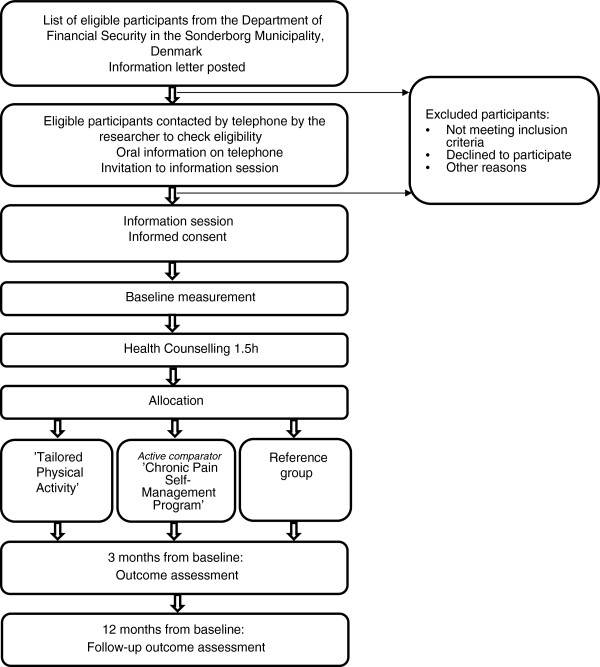
Flow diagram of the progress through the study.

This study is a parallel randomised single-blind controlled trial. It will evaluate the efficacy of TPA including general aerobic training and specific strength training versus REF on return to work. CPSMP will be included as an active comparator as illustrated in Figure 1.

It is being conducted in the Sonderborg Municipality. The trial duration is March 2011 to October 2013.

The study will utilize an allocation concealment process, ensuring that the group to which the participants are allocated is not known before the participant is enrolled in the study.

To monitor the conduct of the study, a project steering group has been appointed. It consists of the participating scientists from the Institute of Sports Science and Clinical Biomechanics at the University of Southern Denmark, the project manager from the Sonderborg Municipality, the head of the Municipality’s rehabilitation team, the head of the training team and four coordinators for the test personnel and each of the three trial interventions.

The protocol is approved by the Regional Scientific Ethics Committee for Southern Denmark (project-ID S-20110040) and The Danish Data Protection Agency. The trial is registered in the ClinicalTrials.gov, number NCT01356784. All of the participants will give written informed consent before enrolment.

### Settings

The participants will be recruited from lists of potential participants provided every second month by the Department of Financial Security in the Sonderborg Municipality. Pre- and post-intervention tests and assessments, in addition to the interventions, will be performed at the Health Care Centre in Sonderborg.

### Study population

The population will consist of citizens from the Sonderborg Municipality who have been sick-listed for a maximum of 9 weeks. The inclusion criteria are: (1) pain related to the back or the upper body, and (2) currently sick-listed for a period of a maximum of 9 weeks.

Some participants will be prevented from participating in the interventions due to part- or full-time return to work or engagement in a rehabilitation programme in another sector e.g. testing for work ability ordered by their job section in the municipality.

Participants will be sequentially recruited in eleven time sequences. At designated time points in the recruitment period the Department of Financial Security will generate a list of potential participants from a database with two main categories of sick leave: (1) shoulder/arm/hand and (2) back/hip/neck. The people with hip problems are excluded at the first personal telephone contact.

The time intervals between the sequences are designed such that citizens who are sick-listed for more than 3 weeks will be asked if they would like to participate in the study. Among those who are sick-listed for a period of less than 9 weeks, not all will be invited to participate in the study because some may have already returned to work within that period. Those sick-listed on the day of generating the list can be included.

Excluded participants or eligible participants who do not want to participate will be registered in one of three categories as recommended by the CONSORT statement: (1) Not meeting the inclusion criteria, (2) Declined to participate, or (3) Other reasons [18].

### Procedure for recruitment, randomisation and allocation

All eligible participants will receive written information including an invitation to an information meeting. After the meeting, the participants will give written informed consent if they agree to enrol in the study.

The recruited participants will be randomised into permuted blocks of 3 and 6, according to computer-generated random numbers, to participate in TPA, CPSMP or REF.

To ensure concealment of the assigned intervention, the receptionist in the Health Care Centre will obtain the opaque, sealed envelope containing the participant’s assigned intervention after the participants have received health guidance and just before the intervention is initiated. Neither the investigator nor the health personnel in the Health Care Centre have any other role in the sequence generation and subsequent allocation concealment.

### Interventions

Participants will be randomised to one of three arms. All randomised participants will receive health guidance for 1.5 hours from a trained supervisor. Additionally, the two intervention groups will be offered health promotion activities. Interventions will start within one week from baseline measurements, health guidance and the randomisation.

*Health guidance* is a 1.5-hour dialogue between the participant and health supervisor, based on the participant’s lifestyle, motivation, resources and power to act. During the conversation the participant will have the opportunity to prepare a goal-oriented health plan identifying the means to achieve the changes that the participant wants and needs. The health supervisor will inspire and support the participants to take an active part in their own lives, such as increasing wellbeing in everyday life, physical activity and weight loss, as well as smoking cessation.

#### Tailored Physical Activity-group (TPA)

This intervention group will receive TPA in addition to health guidance.

TPA sessions will be performed in teams of up to 10 participants and include a standardised combination of aerobic fitness and strength training for 50 minutes 3 times per week over 10 weeks supervised by a physiotherapist at the Health Care Centre in the Sonderborg Municipality. The participants will be referred to one of three standardised training programmes based on their primary region of musculoskeletal problems (neck and shoulder pain, arm and/or hand pain, lower back pain).

The three standardised training programmes all start with 5 minutes warm up during which the participants will gradually increase their heart rate (HR) followed by aerobic fitness training for 20 minutes at intensities ranging from 50% with a progression up to 80% heart rate reserve. During the following weeks, training will be tailored to the participant’s current training status and pain problems [19].

For the warm up and the aerobic fitness training, the participants can choose between ergometer cycling, rowing, stepping or cross training. The choice is taken after consultation with the physiotherapist taking into consideration the participant’s current musculoskeletal troubles and general health. The relative workload will be estimated based on the known relationship between HR and oxygen uptake, i.e. relative workload = (working HR – resting HR)/(maximum HR – resting HR). Resting HR is set at 70 beats per minute and maximum HR is estimated at 208 – (0.7 × age) [20]. HR is monitored during each training session to ensure an optimal training intensity.

Participants with pain that is related to the upper body and the neck are referred to high-intensity strength training in modified programmes [9,10]. The programme for neck and shoulder pain contains five different dumbbell exercises; one-arm row, shoulder abduction, shoulder elevation, reverse flies and upright row. The participants with pain primarily located in the arm and/or hand region will participate in a programme with five different dumbbell exercises: front raise, shoulder abduction, reverse flies, shoulder elevation and wrist extension.

During the intervention period, the training load will progressively be increased from 15 repetitions maximum (~70% of maximal intensity) at the beginning of the training period to 8–12 repetitions maximum (~75-85% of maximal intensity) during the later phase. The strengthening exercises will be performed in a conventional manner using consecutive concentric and eccentric muscle contractions. Three of the five different exercises with three sets per exercise will be performed during each training session in an alternating manner, with shoulder elevation being the only exercise that is performed during each session [9].

Participants with symptoms in the lower spine will be referred to a specific strength training similar to the exercises for the upper body, in addition to coordination exercises for the lower spine. The exercises are standardised and composed of exercises activating the rectus abdominis, erector spinae and oblique externus muscles to more than 60% of their maximal voluntary contraction [16]. The rate of progression of all the exercises will be dependent upon strength gains.

Only physiotherapists educated in accordance with the manuals for the training concepts will take part in the project to ensure standardised guidance and supervision. The physiotherapists will be encouraged to use their professionalism judgment to calibrate each individual’s programme based on the response of their musculoskeletal condition to the physical demands of the programme. The training activity will be recorded in a diary at the end of each session.

#### “Chronic Pain Self-Management Program”-group (CPSMP)

This active comparator group will receive CPSMP in addition to health guidance. CPSMP is developed for people who have a primary or secondary diagnosis of chronic pain. Pain is defined as being chronic or long term if it has lasted for longer than 3 to 6 months.

The CPSMP is a weekly workshop of 2.5 hours for 6 weeks in the Health Care Centre in Sonderborg. Workshops will be facilitated by two trained leaders, at least one of whom is a non-health professional who suffers from chronic pain.

Topics covered in the teaching sessions will include: (1) techniques to deal with problems such as frustration, fatigue, isolation, and poor sleep, (2) appropriate exercise for maintaining and improving strength, flexibility, and endurance, (3) appropriate use of medications, (4) communicating effectively with family, friends, and health professionals, (5) optimal nutrition, 6) pacing activity and rest, and (7) how to evaluate new treatments.

Additionally, there will be homework in the form of working with plans for action and reading a course book. The participants will take part in courses with group sizes of 12–18 persons and the teaching method will be based on the concept of self-efficacy.

The process used during the teaching programme is supposed to be the key element that makes the programme effective. Classes are highly participative and mutual support and success will build the participants’ confidence in their ability to manage their own health, maintain activities and fulfill their life expectations. The CPSMP will not conflict with existing programmes or treatment and is designed to enhance the regular treatment. The programme offers the participants new skills in order to manage initiatives for promoting their health, as well as to help them keep active in their daily life [21].

#### Reference-group (REF)

The REF group will receive health guidance only.

### Outcome measures

Measurements will take place at baseline and after 3 months at the end of the intervention. A secondary follow-up measurement will be performed 12 months after baseline to examine long-term effects. Baseline demographic characteristics of participants will also be recorded.

The *primary endpoint* for efficacy will be the participants’ sick-listed status (yes/no) as registered by the Department of Financial Security in the Sonderborg Municipality. As a *co-primary endpoint,* the duration of the sickness absence within the evaluation period will be examined.

Secondary endpoints will include anthropometric measures, hand-grip strength and aerobic capacity. In addition, we will evaluate musculoskeletal symptoms, general health, work ability, physical capacity, kinesiophobia, physical functional status, interpersonal problems and mental disorders, via self-reported questionnaires.

*Anthropometric measurements* will be performed by trained physiotherapists. Weight, height and circumference of waist and hip will be measured.

*Hand-grip strength* will be measured in kilograms with a digital hand-held dynamometer. Participants will be instructed to sit upright on a chair with the safety strap around their wrist, with their arm at right angles and their elbow by the side of their body. Wrist extension only up to 30° will be allowed. The participants will be strongly encouraged to squeeze with maximum effort. Three trials will be recorded and an extra trial will be conducted if force is changed more than three kilograms compared with a previous attempt [22].

*Aerobic capacity* will be estimated with the Aastrand-Rhyming Test, which is a submaximal cycle ergometer aerobic fitness test. The participants will cycle 60 rpm at a work load set at a level referenced to the sex and condition of the subject. The participant’s HR is measured during the exercise and the test will be terminated when the subject reaches a steady state HR of between 120 and 160 beats/min, with a change of less than 5 beats between two consecutive minutes. Aerobic capacity will be estimated based on Aastrands nomogram, using the participant’s work-load and HR during testing [23]. Finally, the result will be adjusted for age and gender and normalised to body weight.

*Musculoskeletal symptoms* in the shoulder, elbow, hand, neck, upper back and lower back will be evaluated with a modified version of the Nordic Musculoskeletal Questionnaire. The questions used are “Have you, at any time during the last 3 months had trouble (such as ache, pain, discomfort) in [body part]?” (yes/no), “How many days have you had trouble (such as ache, pain, discomfort) in [body part] during the last 3 months?” (0 days, 1–7 days, 8–30 days, 30 days but not every day, every day), “How many days in total have you been on sick leave because of trouble (such as ache, pain, discomfort) in [body part] during the last 3 months?” (0 days, 1–7 days, 8–30 days, 30 days), “Because of trouble (such as ache, pain, discomfort) in [body part] have you been examined or treated by a doctor, chiropractor or physiotherapist or the like during the last 3 months (yes/no), “Have you had trouble (such as ache, pain, discomfort) in [body part] during the last 7 days?” (yes/no). Illustrations from the Nordic Questionnaire define the respective body regions of the neck, right shoulder, left shoulder, upper spine, lower spine, right elbow, left elbow, right hand and left hand [24].

*General health and health-related quality of life* will be measured using the SF-36 Health Survey, a standardised questionnaire investigating eight health concepts: physical functioning, role limitations because of physical functioning, bodily pain, general health, vitality, social functioning, role limitation because of emotional problems and mental health. Answers are recorded using a Likert scale [25,26].

*Work ability* will be assessed by the single-item measure that was originally part of the widely used Workability Index. However, recent studies have shown that the single item question is a reliable and easy tool with validity comparable with the full index [27]. The question used is “Imagine that your work ability is worth 10 points when it is at its best. How many points would you give your present work ability?” A numerical rating scale was used where 0 represents “not able to work” and 10 represents “the highest work ability” [28].

*Kinesiophobia* are dysfunctional beliefs about physical activities that will be assessed using the Tampa Scale for Kinesiophobia. It is a 17-item questionnaire to assess fear of (re)injury due to movement, because avoidance behavior can be one mechanism in sustaining chronic pain disability. Each item is provided with a 4-point Likert scale with scoring ranging from “strongly agree” to “strongly disagree” [29-31].

*Perceived disability* in terms of self-reported activity limitation for the primary region of pain will be measured by the Neck Disability Index (NDI), Disabilities of the Arm, Shoulder or Hand (DASH) or Roland Morris Disability Questionnaire (RMQ).

The NDI is a 10-item questionnaire designed to measure disability in activities of daily living due to neck pain. Each item has 6 response options ranging from no pain and no functional limitation to worst pain and maximal limitation [32,33].

The DASH is a 30-item questionnaire with five response options for each item. It is designed to measure physical function and symptoms for musculoskeletal disorders of the upper limb [34,35].

The RMQ is a 23-item questionnaire that assesses the degree of functionality and disability due to low back pain and/or sciatica. Each item is scaled yes/no. ‘No’ corresponds to no disability and ‘yes’ corresponds to self-rated disability on each item [36].

*Mental Disorders* will be assessed by the Screening Questionnaire of Common Mental Disorders (CMD-SQ) consisting of 37 items in validated subscales (SCL-SOM, Whiteley-7, SCL-ANX-4, SCL-8, SCL-DEF-6, CAGE) measuring anxiety, depression, use of alcohol, and somatisation. The patients respond on a five-point Likert scale [37-40].

*Interpersonal problems* will be assessed by the IIP (Inventory of Interpersonal Problems). The measurement of interpersonal problems allows a differentiation of interpersonal and non-interpersonal sources of distress (e.g. depressed mood, anxiety)*.* The IIP (short form) consists of 64 items scored on eight scales. The scales include areas that may be difficult for a person to do and areas that indicate things where a person may do too much. The eight scales (domineering, vindictive, cold, socially inhibited, non-assertive, exploitable, overly nurturing and intrusive) are scored on a five-point Likert scale [41,42].

*Self-assessed physical fitness* will be evaluated with a questionnaire based on Stroyer et al. but modified from a VAS-scale to a Likert scale [43]. It consists of five items with illustrations of five situations reflecting aerobic fitness, muscle strength, endurance, flexibility and balance. The participants will be asked “How would you score the following components of physical fitness compared with others of your own age and sex?”. A Likert scale will be used and one represents “poor” or “weak” and 10 represents “good” or “strong”.

### Blinding

Health care professionals and participants will be aware of the allocation arm but blinded to the results of any former assessment. Health care professionals who are outcome assessors will be blinded to participants’ allocation.

### Sample size

Assuming 70% or more of the participants in the TPA group and 43% of the participants (estimate based on previous data) in the REF group would return to work during the 3-month period of observation, a sample size of 46 individuals in each group will be required to achieve greater than 80% statistical power (two-sided, alpha = 0.05). The eleven time sequences of enrollment are required to recruit this number of participants.

Prospectively, this study will not be powered with a pre-hoc assumption of superiority or non-inferiority between the TPA and CPSMP groups. The CPSMP group is included as an active comparator.

### Statistical analysis

All participants who receive a group allocation will be included for efficacy and feasibility (intention-to-treat population). The primary outcome, differences across the groups in the proportion of participants who are not sick-listed at 3 and 12 months, will be calculated using a Chi-squared test (and 95 confidence intervals). Also, the rate of return to work at 3 months (co-primary outcome measure), will be calculated based on the number of days with sickness absence as recorded by the Department of Financial Security in Sonderborg Municipality. We will compare the time on sick leave across the groups using the Kaplan-Meier method and report the median time on sick leave with a 95% confidence interval. A Cox proportional hazard model will be used to measure the effectiveness of TPA and CPSMP relative to REF, which will be reported as hazard rate ratio (HRR) with a 95% confidence interval. Participant characteristics that vary amongst the three groups at baseline will be included in a multivariate Cox proportional hazard model to control for their potential confounding effect.

## Discussion

This study will contribute to evidence-based recommendations regarding initiatives for citizens who are sick-listed due to pain that is related to the back or the upper body.

Rehabilitation of people with work disability is a major challenge for municipalities when pain causes loss of function and the citizens receive sickness benefits because they cannot maintain their participation in the labor market. Pain-related sick leave amongst younger workers, is often based on symptoms that are rarely consistent with a specific disease [2].

After the Danish Local Government Reform in 2007, and embodied in the Health Act, the patient- and citizen-oriented rehabilitation is a joint responsibility of Denmark’s 98 municipalities and 5 regions and it is an important aspect of strengthening prevention of long-term work disability. Besides the 71 municipalities in Denmark which offer general patient education programmes developed by the Stanford University School of Medicine, there are other methods of group-based patient education that are widely used that also involve health care professionals as teachers [14]. But the organisation of patient education is complicated by the effects of such programs being largely unknown [15]. On the other hand, preventive strategies in the workplace aimed at enhancing physical capacity and/or the ability to cope with musculoskeletal pain have been successfully tested [9,10,16,17,44,45] but their efficacy with respect to return to work has not been investigated. Accordingly, the municipalities will be the primary beneficiaries of the results from this investigation of intervention programmes for sick-listed citizens.

## Competing interests

The authors declare that they have no competing interests.

## Authors’ contributions

LNA, TLS and KS initially designed the study. All authors contributed to developing the protocols and intervention materials. LNA and KS drafted the manuscript and all authors were involved in revising it for intellectual content and have given final approval of the version to be published. All authors read and approved the final manuscript.

## Pre-publication history

The pre-publication history for this paper can be accessed here:

http://www.biomedcentral.com/1471-2458/13/66/prepub
